# De novo transcriptome analysis of *Lantana camara* L. revealed candidate genes involved in phenylpropanoid biosynthesis pathway

**DOI:** 10.1038/s41598-020-70635-5

**Published:** 2020-08-13

**Authors:** Muzammil Shah, Hesham F. Alharby, Khalid Rehman Hakeem, Niaz Ali, Inayat Ur Rahman, Mohd Munawar, Yasir Anwar

**Affiliations:** 1 Department of Biological Sciences, Faculty of Science, King Abdulaziz University, Jeddah, 21589 Saudi Arabia; 2 Department of Botany, Hazara University, Mansehra, KP 21300 Pakistan; 3 William L. Brown Center, Missouri Botanical Garden, P.O. Box 299, St. Louis, MO 63166-0299 USA

**Keywords:** RNA sequencing, Plant genetics

## Abstract

*Lantana camara* L. is an economically important essential oil producing plant belonging to family Verbenaceae. It is used in medication for treating various diseases like cancer, ulcers, tumor, asthma and fever. The plant is a useful source of essential bioactive compounds such as steroids, flavonoids and phenylpropanoid glycosides etc. Nonetheless, very little is known about the genomic or transcriptomic resources of *L. camara*, and this might be the reason of hindering molecular studies leading to identification of improved lines. Here we used Illumina sequencing platform and performed the *L. camara* leaf (LCL) and root (LCR) de novo transcriptome analyses*.* A total of 70,155,594 and 84,263,224 clean reads were obtained and de novo assembly generated 72,877 and 513,985 unigenes from leaf (LCL) and root (LCR) respectively. Furthermore, the pathway analysis revealed the presence of 229 and 943 genes involved in the phenylpropanoid biosynthesis in leaf and root tissues respectively. Similarity search was performed against publically available genome databases and best matches were found with *Sesamum indicum* (67.5%) that were much higher than that of *Arabidopsis thaliana* (3.9%). To the best of our knowledge, this is the first comprehensive transcriptomic analysis of leaf and root tissues of this non-model plant from family Verbenaceae and may serve as a baseline for further molecular studies.

## Introduction

*Lantana camara* L. belonging to family Verbenaceae is an evergreen shrub, native to the Neotropics and grown worldwide for its medicinal and ornamental value^1^. The plant may grow up to 4 m, forming dense thickets^2^ and is extensively used in traditional herbal medicines of many cultures including Saudi Arabia^3^. The medicinal properties of *L. camara* are attributed to the presence of many bioactive compounds with therapeutic potential such as steroids, flavonoids, triterpenoids, oligosaccharides, iridoide glycosides, naphthoquinones and phenylpropanoid glycosides^4–6^. Furthermore, *L. camara* is used in medication for treating cancer, ulcers, tumors, tetanus, cuts, eczema, measles, chicken pox, fevers, rheumatism and asthma^1,7,8^. Several important phytochemicals have been isolated from *L. camara* including ursolic acid, oleanolic acid, linaroside, lantanoside, verbascoside, camarinic acid, phytol and umuhengerin etc. and their biological activities such as anticancer, antibacterial, antioxidant, antiulcer and nematocidal have been reported^9–11^. Nonetheless, *L. camara* is also known as a major source of easily available plant essential oil known as Lantana oil^12,13^. Literature survey indicates the substantial diversity in the composition of essential oils isolated from *L. camara* growing in diverse localities^14–18^.

In spite, much is known about the phytochemistry, toxicology and medicinal properties of *L. camara*^19^ very little is known about the genomic architecture of the plant. To date, only 41 sequences including (rps3, atpB, ccsA, rpoC1, rpoC2, FT, GLO1, rpl32 and rbcL) have been deposited to the NCBI Genbank database^20^. To stop the spread of *L. camara* as an invasive weed, transcriptomes of young ovaries were recently studied and possible mechanisms of unreduced female gametes formation were elaborated^21^. Complete genome sequences provide invaluable insights into the biological functions of individual genes and proteins and therefore, hold immense promise for crop improvement and breeding of new cultivars. The genome of *L. camara* has not been sequenced yet and no details are available on the genomics or transcriptomics of the plant. Therefore, a detailed research strategy is needed to elucidate various aspects of genomics and transcriptomics of this important medicinal plant. Recently we have also reported the complete chloroplast genome of *L.camara*^22^*.* Nowadays, transcriptomics using next generation sequencing (NGS) technology has emerged to be one of the most powerful and cost-effective approaches generating huge data on transcribed sequences that may be implemented in wide research purposes^23^. The practical applications of transcriptome sequencing have been utilized in various medicinal plants as well as crop plants including rice^24^, maize^25^, chickpea^26^, wheat^27^, *Camelina sativa*^28^, *Quercus pubescens*^29^, finger millet^30^, *Stellera chamaejasme*^31^ and *Aconitum heterophyllum*^32^. As a step forward, here we report the first de novo transcriptome assembly of *L. camara* leaf and root tissues that may lay the foundation for rapid identification of functional genes discovery and genomics-assisted breeding for developing more efficient *L. camara* lines with desired traits.

## Materials and methods

### Plant material and RNA quantification

Plants were grown in the laboratory of plant physiology King Abdulaziz University, Jeddah, Saudi Arabia under 28 °C/22 °C day and night temperature in a semi-controlled green house. No specific permits were required for the described study. Fresh leaf (LCL) and root (LCR) tissues were harvested from 02 months old plant, washed thoroughly with sterile water, and immediately frozen in liquid nitrogen. RNA isolation was carried out using RNeasy Plus Mini Kit (Qiagen, Cat. No: 74134). RNA degradation and contamination were monitored on 1% agarose gel. Purity of RNA was checked on NanoPhotometer^®^ spectrophotometer (IMPLEN, CA, USA). Quantification and integrity of RNA was assessed using the RNA Nano 6000 Assay Kit (Agilent Technologies, CA, USA).

### Library preparation for transcriptome sequencing

NEBNext^®^ Ultra™ RNA Library Prep Kit was used to generate sequencing libraries. Briefly, mRNA was purified from total RNA using poly-T oligo-attached magnetic beads. Divalent cations were used to carry out fragmentation under high temperature in NEBNext (FSSR) first strand synthesis reaction Buffer (5×). Random hexamer primers were used to synthesize first strand cDNA using M-MuLV Reverse Transcriptase (RNase H-). Second strand cDNA synthesis was carried out using DNA Polymerase I and RNase H. Exonuclease/polymerase activity was performed to convert remaining overhangs into blunt ends. Ligation of NEBNext adaptor with hairpin loop was performed after adenylation of 3′end of DNA fragments in order to prepare sample for hybridization. The cDNA fragments of 250–300 bp were preferentially selected and purification of the library fragments was carried out with AMPure XP system (Beckman Coulter, Beverly, USA). 3 µl of Enzyme (NEB, USA) was used with size-selected, adaptor-ligated cDNA at 37 °C for 15 min, followed by 5 min at 95 °C. PCR was carried out with Phusion High-Fidelity DNA polymerase, Universal PCR primers and Index (X) Primer. PCR products were purified (AMPure XP system) and library quality was assessed on the Agilent Bioanalyzer 2100 system. Paired-end library of 150 bp (PE150) was prepared following Illumina protocol/instructions.

### Clustering, quality control and transcriptome assembly

Cluster generation system (PE Cluster Kit cBot-HS Illumina) was used to perform clustering of the index coded samples following manufacturer’s instructions. The library preparations were sequenced on an Illumina platform after cluster generation and paired-end reads were generated. Transcriptome assembly was performed using Trinity software^33^ with min_kmer_cov set to 2, and all other parameters with default settings.

### Gene functional annotation and biological pathways assignment

Libraries of LCL and LCR tissues were annotated using BLASTX against the NCBI database and all unigenes were applied for homology searches. Gene functional annotation was performed using the following 7 databases: NCBI non-redundant protein sequences (NR), NCBI non-redundant nucleotide sequences (Nt), Protein family (Pfam), Clusters of Orthologous Groups of proteins (KOG/COG), manually annotated and reviewed protein sequence database (Swiss-Prot), KEGG Ortholog database (KO)^34^ and Gene Ontology (GO) database. Best aligning results were selected to annotate the unigenes; if aligning results of these databases were not similar, NR database results were preferentially selected**.** For assignment of function to unigenes, Gene Ontology (GO) enrichment analysis was performed using AgriGO (https://bioinfo.cau.edu.cn/agriGO/analysis.php)^35^ and annotated sequence/s may have more than one GO term. Similarly, to understand the high-level functions and gain an overview of gene pathway networks KEGG^36,37^ was used. Enzyme commission (EC) numbers were assigned to unique sequences, based on the BLASTx search of protein databases, using a cutoff E value 10 − 5. The statistical enrichment of DEGs in KEGG pathways was carried out using KOBAS^38,39^.

### Quantification and differential expression analysis

The RNA-seq by Expectation Maximization (RSEM) package was used to estimate gene expression levels of both root and leaf tissues^40^. At first clean data was mapped back onto the assembled transcriptome and then read count for each gene was obtained from the mapping results. Prior to differential gene expression (DEGs) analysis of the sequenced library of root and leaf tissues, read counts were adjusted through one scaling normalized factor and analysis was performed using DEGseq (2010) R package^41^. P value was adjusted using q value of < 0.005 and |log2(foldchange)| > 1 was set as the threshold for significantly differential expression. To identify DEGs between the LCR and LCL datasets, raw read counts were initially filtered to exclude orphan transcripts. Pairwise comparison was performed using the default parameters of DEGseq (2010) R package and an adaptive t shrinkage estimator was followed for ranking and visualization of log-fold changes (log2FC, LFC) of the DEGs^42^.

## Results and discussion

### RNA sequencing and de novo assembly

Advances in genomics have led to the development of NGS based trait mapping approaches that have tremendously increased the efficiency of traits selection and mapping in complex genomes^43^. Among the high throughput genotyping approaches, Next Generation RNA Sequencing technology has contributed to a more comprehensive understanding of functional genes and is widely used for characterization of transcriptome profiles of various model and non-model plants^44^. De novo transcriptome analysis is not only providing an excellent platform for finding novel genes, molecular markers development but also cater a base for the construction of networks of gene expressions for various tissues and organs of animals as well as plants. Although, the number of available high-quality reference genomes has been constantly growing still, de novo transcriptome approaches are mainly used for non-model species where whole genomes information are missing. The remarkable advances in RNA sequencing provide a cost-effective way to obtain large amounts of transcriptome data from many organisms and tissue types, especially in the complete absence of a reference genome thereby, allowing us to identify all expressed transcripts^33,34^. Here we provide, the first report on transcriptome profiling and DEGs of *L. camara* leaf and root tissues; RNA-Seq library was constructed and RNA samples with RIN value more than 6 were used (Supplementary file 1). A total of 76,315,644 and 87,218,768 raw reads were obtained for LCL and LCR respectively. For the removal of adapters, poly-A tail, primer sequences and short as well as low quality sequences trimming process was performed that resulted in a total of 70,155,594 clean reads from LCL and 84,263,224 from LCR. The total size of clean bases generated was 10.5 GB for LCL sample with percent error (0.01%), Q20 (97.39%), Q30 (93.47%) and GC content (45.61%), whereas for LCR sample 12.6 GB with a percent error (0.03%), Q20 (96.81%), Q30 (92.01%) and GC content (43.73%) (Table 1). The sequences (raw data) generated were deposited to NCBI as PRJNA503321 (for leaf sample) and PRJNA605469 (for root sample). For samples lacking reference genomes, clean reads need to be assembled to get a reference sequence. We used Trinity assembler^33^ to get the leaf and root transcriptome information.

**Table 1:** Quality of reads obtained after RNA-sequencing of leaf and root transcriptomes.

Samples	Raw reads	Clean reads	Clean bases (GB)	Error (%)	Q20 (%)	Q30 (%)	GC (%)
Leaf	76,315,644	70,155,594	10.5	0.01	97.39	93.47	45.61
Root	87,218,768	84,263,224	12.6	0.03	96.81	92.01	43.73

A total of 72,898 and 516,026 transcripts were obtained from LCL and LCR respectively and length of transcripts varied for leaf (201 to 14,753 bp) and root (201 to 16,796 bp) (Table 2). A total 72,877 unigenes were detected in LCL tissues of which 19,955 were within 200–500 bp; 21,816 within 500–1kbp; 20,062 within 1 k–2 kbp and 11,044 were > 2 kbp, with an N50 of 1,650 bp (Table 2). Relatively high number *i.e.* 513,985 of unigenes were identified in LCR tissue, of which 320,891 were within 200–500 bp; 107,176 within 500-1kbp; 59,405 within 1 k-2 kbp and 26,513 were within > 2 kbp, with an N50 of 939 bp (Table 2).

**Table 2:** Summary of transcripts length distribution and unigenes assembled.

Length interval	Leaf		Root	
	No of transcripts	No of unigenes	No of transcripts	No of unigenes
200–500 bp	19,976	19,955	322,930	320,891
500–1 kbp	21,816	21,816	107,178	107,176
1 k–2 kbp	20,062	20,062	59,405	59,405
> 2 kbp	11,044	11,044	26,513	26,513
Total	72,898	72,877	516,026	513,985
Minimum length	201	201	201	201
Maximum length	14,753	14,753	16,796	16,796
N50	1,650	1,650	937	939
N90	541	541	279	280
Total nucleotides	83,817,239	83,812,099	336,871,604	336,370,588

### Functional annotation of genes

Unigenes of the LCL and LCR tissues were annotated using BLAST search against the NR, NT, PFAM, GO and KOG databases. Gene Ontology assignments based on the protein match and annotation results are based on Götz et al*.*^45^ Of the 72,877 and 513,985 unique transcript sequences of LCL and LCR annotated, a large proportion of the sequences (73.27% and 70.11%) had hits in databases. Of the LCL tissue, a total of 50,540 (69.34%) unigenes matched with known proteins in the NR database, while 38,975 (53.48%) unigenes were annotated with entries in the Swiss-prot database, 36,209 (49.68%) unigenes matched with proteins in Gene Ontology (GO) database, 35,711 (49%) matched with proteins in the PFAM database (Table 3). Similarly, the LCR transcriptome revealed that 253,381 (49.29%) of unigenes matched with proteins in the NR database, 250,499 (48.73%) unigenes matched with entries in the Swiss-prot database, 255,964 (49.79%) unigenes matched with proteins in Gene Ontology (GO) database, and 252,005 (49.02%) were annotated with proteins in the PFAM database (Table 3). Of the assembled unigenes, only 9,849 (13.51%) and 45,051 (8.76%) in LCL and LCR respectively, were successfully annotated in all databases. Reasons for the non-annotated sequences could be, lack of conserved protein domains in short sequences, or in some cases the transcriptome have non-coding genes, UTRs, random transcriptional noise or incomplete spliced introns which are non-homologues to the sequences available in the public databases. This may also be one of the possible reasons that genes have not shown expression at the time of RNA extraction or those genes are expressed at very low levels^46^.

**Table 3:** The ratio of successfully annotated genes.

Annotated databases	Leaf		Root	
	No of unigenes	Percentage (%)	No of unigenes	Percentage (%)
NR	50,540	69.34	253,381	49.29
NT	36,890	50.61	177,562	34.54
KO	20,506	28.13	124,076	24.14
SwissProt	38,975	53.48	250,499	48.73
PFAM	35,711	49	252,005	49.02
GO	36,209	49.68	255,964	49.79
KOG	19,818	27.19	155,979	27.44
All databases	9,849	13.51	45,051	8.76
At least one database	53,397	73.27	360,397	70.11
Total unigenes	72,877	100	513,985	100

**Figure 1:**
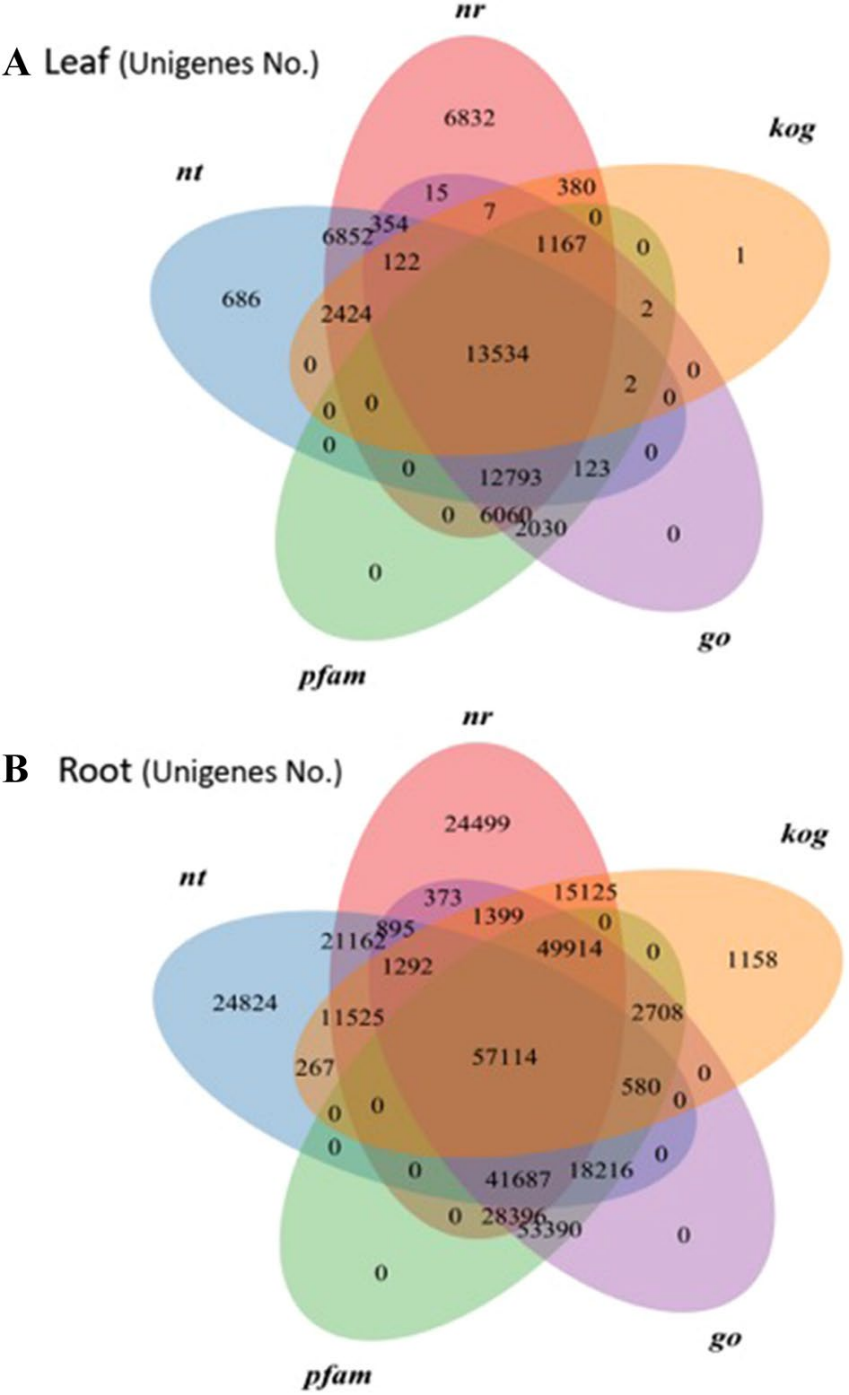
Venn diagram mapping with database annotation.

The assembled unigenes annotation of LCL and LCR tissues is given as Venn diagram (Fig. 1) against PFAM, GO, KOG, NR and NT databases. For leaf tissues, a total of 13,534 unigenes were annotated in 5 databases; 6,060 unigenes sequences showed homology in 3 databases including PFAM, NR and GO. While 1,167 unigenes were annotated in KOG as well as the above three databases and 2,030 unigenes were annotated in PFAM and GO databases (Fig. 1A). Likewise for root tissues, a total of 57,114 unigenes were annotated in five databases where, 28,396 unigenes were homologous in PFAM, NR and GO databases (Fig. 1B).

### Annotation in NR database

**Figure 2:**
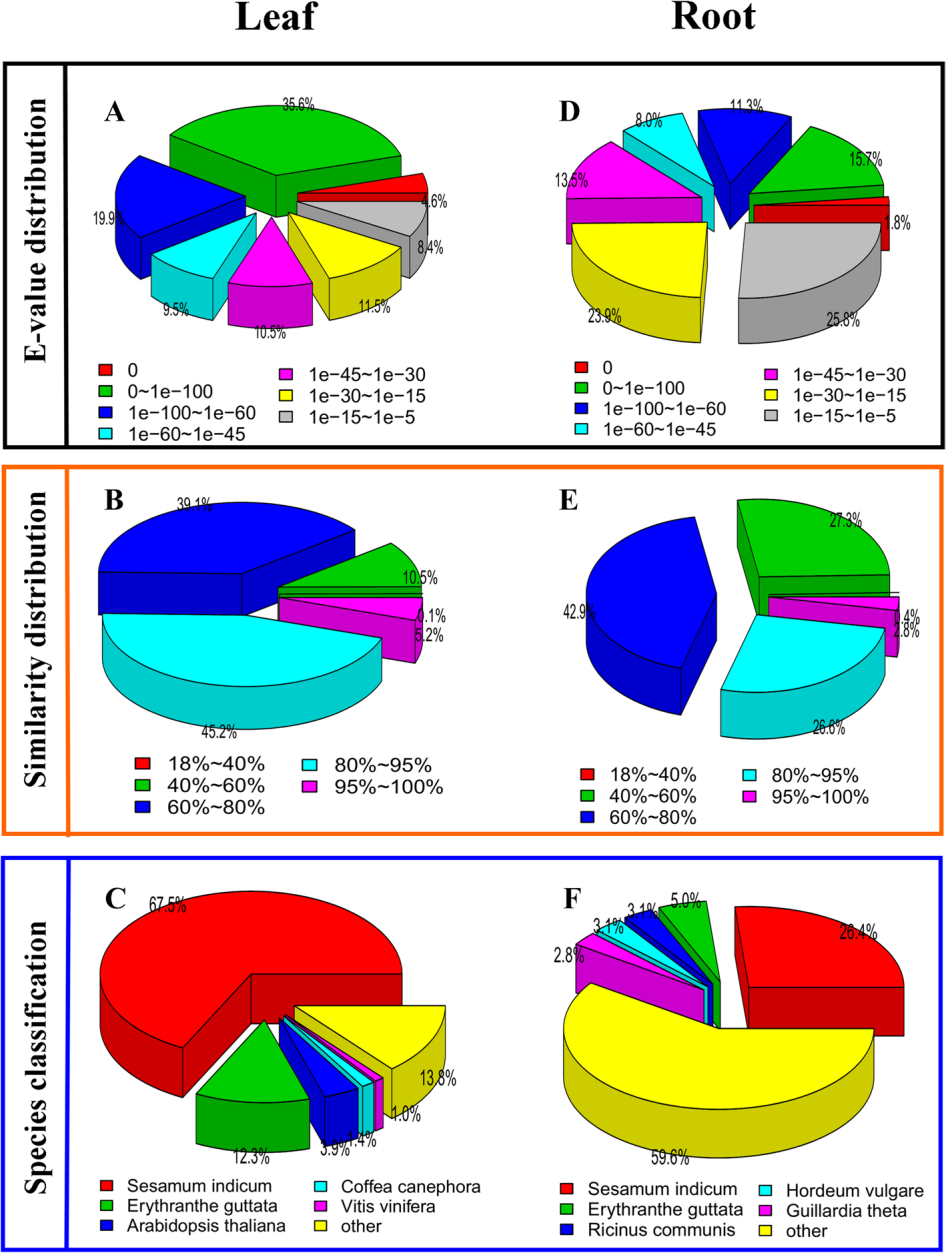
E-value distribution (**A**), similarity distribution (**B**) and species distribution (**C**).

After the high annotation hit score was ascertained in NR database, we considered identifying homologues features of BlastX hits for the annotated unigenes. The E-value distribution in leaf tissue revealed that 35.6% of the annotated unigenes had E-value of 0-1e-100, followed by 19.9% unigenes with 1e−100 to 1e−60 E-value (Fig. 2A). Similarity distribution showed that 45.2% of the unigenes had 80–90% similarities. Furthermore, 39.1% of the unigenes had 60–80% and 5.2% had 95–100% similarities (Fig. 2B). Additionally, comparison of the unigenes annotated with homologous sequences of other plant species was also performed^44^. Among the nucleotide sequences, highest similarity was noted for *Sesamum indicum* (with the highest similarity score of 67.5%). Other species with sequence homology below 13% included *Erythranthe guttata* (12.3%), *Arabidopsis thaliana* (3.9%), *Coffea canephora* (1.4%) and *Vitis vinifera* (1.0%) (Fig. 2C).

E-value distributions in root tissue revealed that 25.8% of the annotated unigenes had E-value of 1e−15 to 1e−5, followed by 23.9% unigenes with 1e−30 to 1e−15 E-value (Fig. 2D). Similarity distribution revealed that 2.8% of the unigenes had 95–100% similarities, 26.6% had 80–95% and 42.9% unigenes had 60–80% similarities (Fig. 2E). Comparison of the annotated unigenes with the homologous sequences revealed highest similarity with *Sesamum indicum* (26.4%) followed by *Erythranthe guttata* (5%), *Ricinus communis* (3.1%) and *Guillardia theta* (2.8%) (Fig. 2F).

### Gene ontology (GO) classification

**Figure 3:**
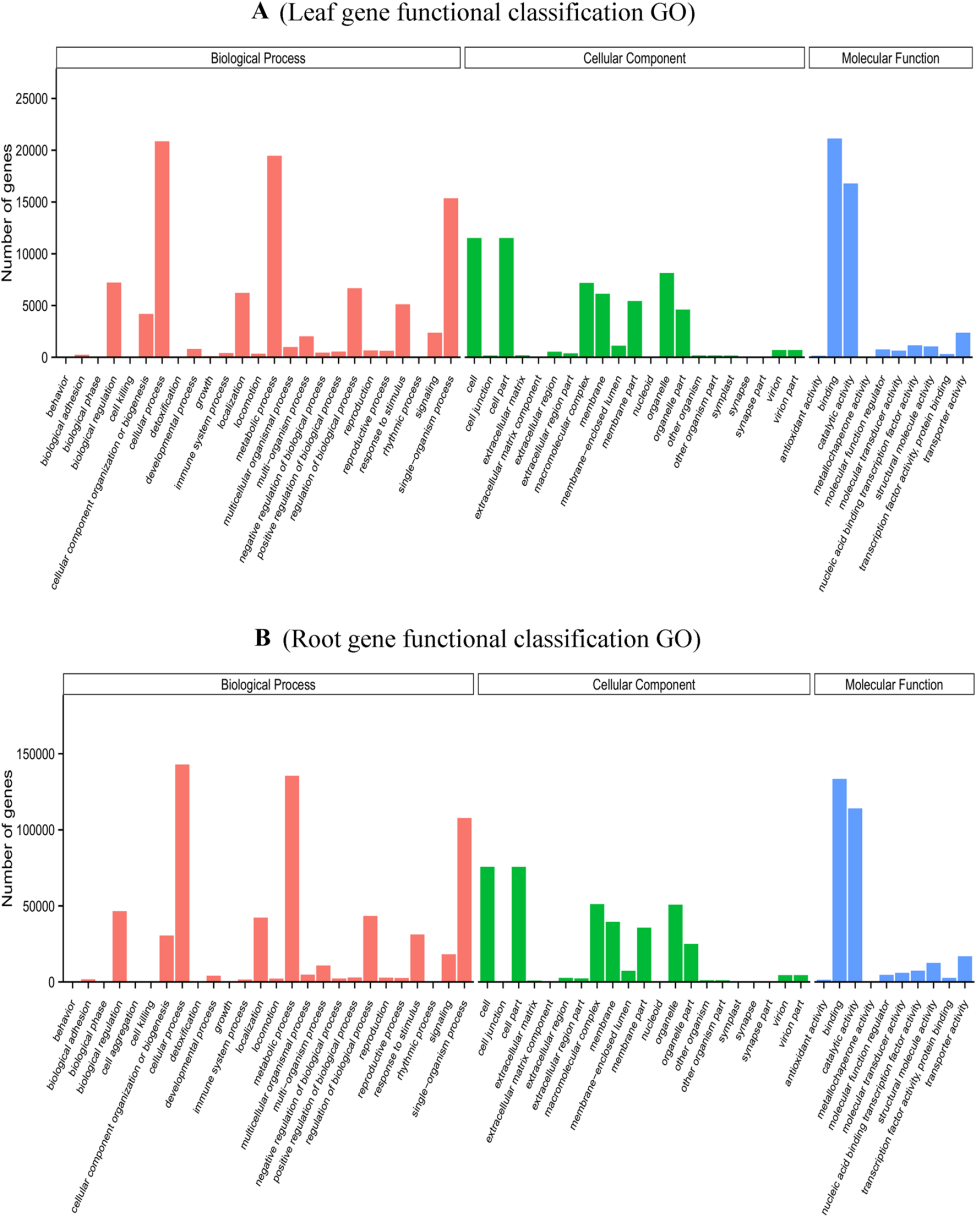
X-axis is the GO term under the three main GO domains; Y-axis is the number and percentage of the annotated genes in the term (sub-term included).

GO assignments were used to classify the functions of the LCL and LCR unigenes, which classified unigenes under the categories of biological process (BP), molecular function (MF) and cellular component (CC). Sum of 36,209 LCL unigenes were classified into three major categories (Fig. 3A). The biological process category, the unique sequences were classified into 25 groups. The most characterized biological processes were ‘cellular process’ (57.59%, GO-ID: 0009987) followed by ‘metabolic process’ (53.72%, GO-ID: 0008152). The cellular components were divided into 21 groups. Interestingly, we found both the represented cellular components ‘Cell’ (31.80%, GO-ID: 0005623) and ‘Cell part’’ (31.80%, GO: 0044464) were similar to previous report^45^. The molecular functions category, the unique sequences clustered into 10 classes. Where, the highest sub-category was ‘binding’ (58.35%, GO-ID: 0005488) followed by ‘catalytic activity’ (46.37%, GO: 0003824) (Fig. 3A, Supplementary file 2).

Similarly, 255,964 LCR unigenes were annotated and also likewise classified into three main categories and further divided into 57 sub-groups (Fig. 3B). Among the biological processes classification, the highest was cellular process 142,929 (55.83%), followed by metabolic process 135,458 (52.92%) and single-organism process 107,769 (42.1%). Furthermore, cellular component was classified in 22 groups, among them high amount of unigenes were related to “cell” 75,691 (29.57%) and “cell part” 75,607 (29.53%), followed by “organelle” 50,774 (19.83%) and “membrane” 35,704 (13.94%) (Fig. 3B, Supplementary file 2). Only few unigenes were assigned to extracellular matrix, extracellular region and extra cellular region part. In the molecular function category, the majority of the unigenes were assigned to “binding” 133,482 (52.14%) and “catalytic activity” 114,050 (44.55%). The annotation and sequence information from Gene Ontology results provide important gene sources for future molecular level studies that underline selection and improvements of *L. camara.*

### KOG classification

**Figure 4:**
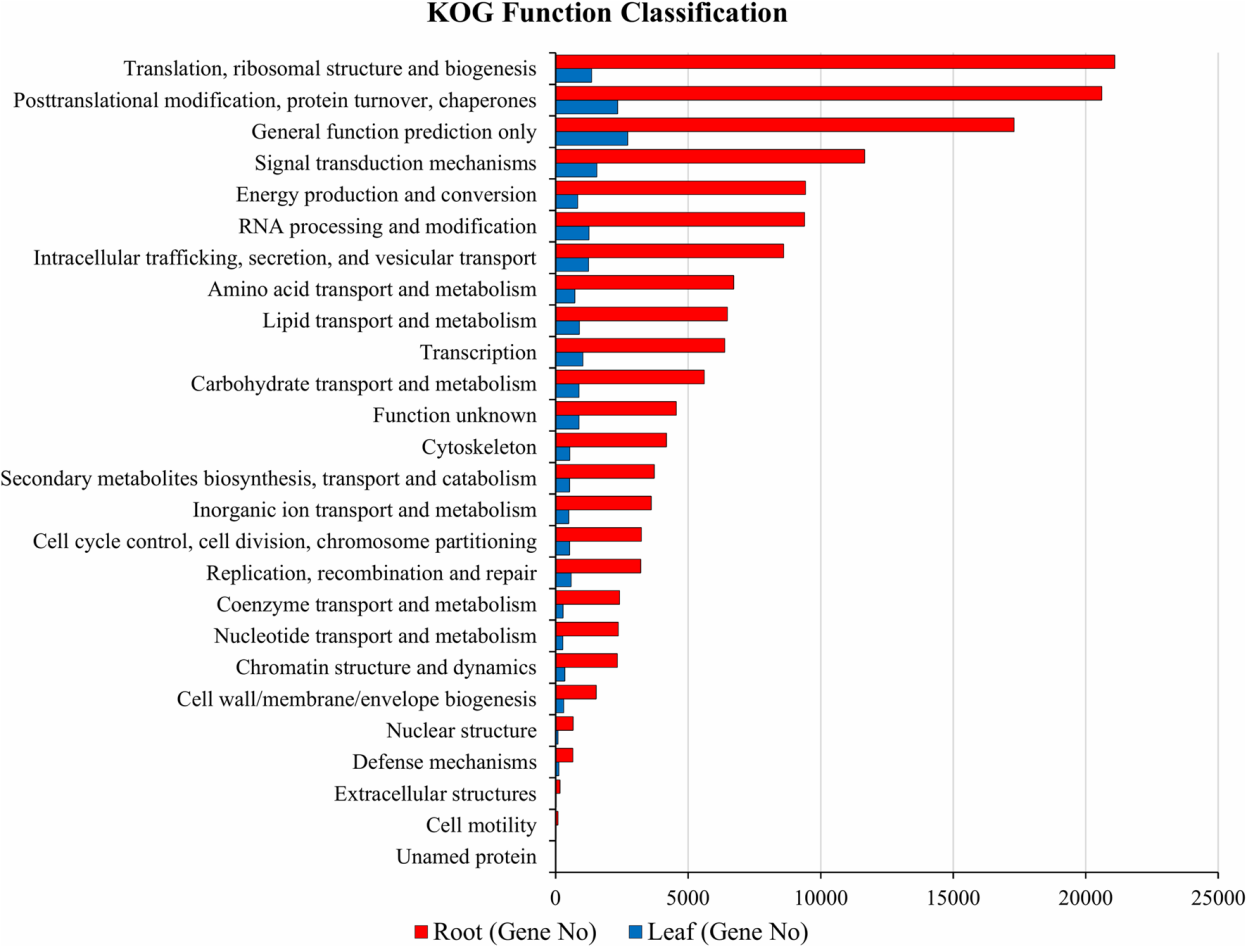
X-axis: names of the 26 KOG group; Y-axis: percentage of annotated genes under this group in the total annotated genes.

On the basis of conserved domain alignment, the annotated unigenes were searched against the KOG database to find the functionally classified orthologous gene products. A total of 19,818 and 155,979 identified genes were annotated in LCL and LCR tissues respectively and these were divided into 26 protein group families (Fig. 4). Among these protein families predicted in LCL tissues, general function prediction (2,719 unigenes, 15.41%) was identified as the highest annotated group, followed by post translational modification, protein turnover, chaperones (2,335 unigenes, 13.23%), signal transduction mechanisms (1,557 unigenes, 8.82%), translation, ribosomal structure and biogenesis (1,355 unigenes, 7.68%), RNA processing and modification (1,257 unigenes, 7.12%), intracellular trafficking, secretion, and vesicular transport (1,241, 7.03%), whereas the smallest groups were nuclear structure (89), extracellular structures (26) and cell motility (15) (Supplementary file 3). In LCR tissues, “Translation, ribosomal structure and biogenesis” consisted the largest category with 21,097 genes (14.95%), followed by “Posttranslational modification, protein turnover, chaperones” with 20,612 genes (14.60%) and “General function prediction only” 17,296 genes (12.25%). These results are not in alignment to previous report where the authors found “General function prediction only” as the highest category with 1,006 (13.86%) genes^47^. Fewer genes were assigned to “cell motility” (92) and unnamed (4) proteins (Fig. 4, Supplementary file 3).

### Functional characterization using KEGG

**Figure 5:**
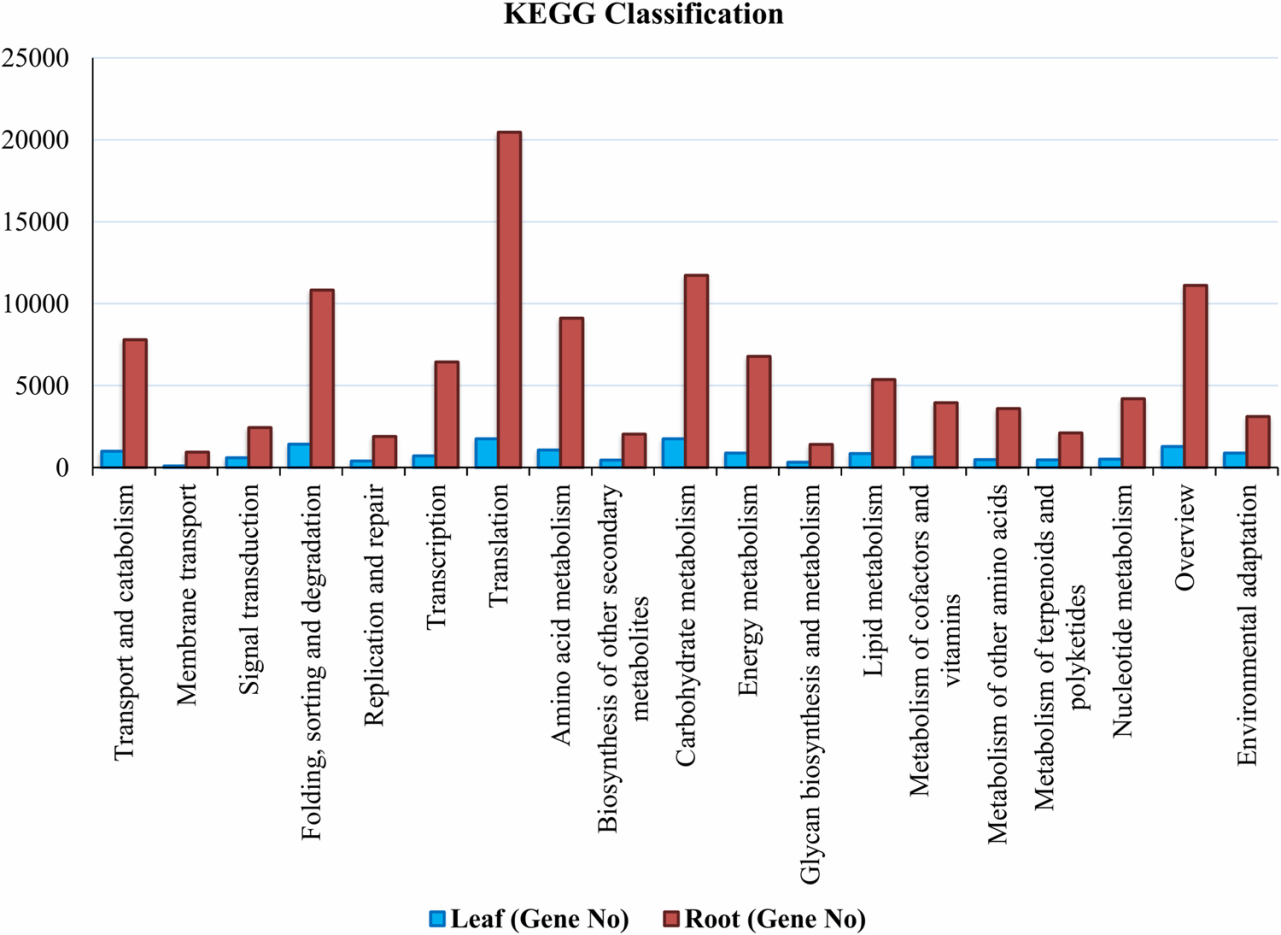
Y-axis: names of KEGG pathways; X-axis is the number of the genes annotated in the pathway and the ratio between the number in this pathway and the total number of annotated genes. The KEGG metabolic pathways gene involved in are divided into five branches; (**A**) Cellular Processes, (**B**) Environmental Information Processing, (**C**) Genetic Information Processing, (**D**) Metabolism and (**E**) Organismal Systems.

**Figure 6:**
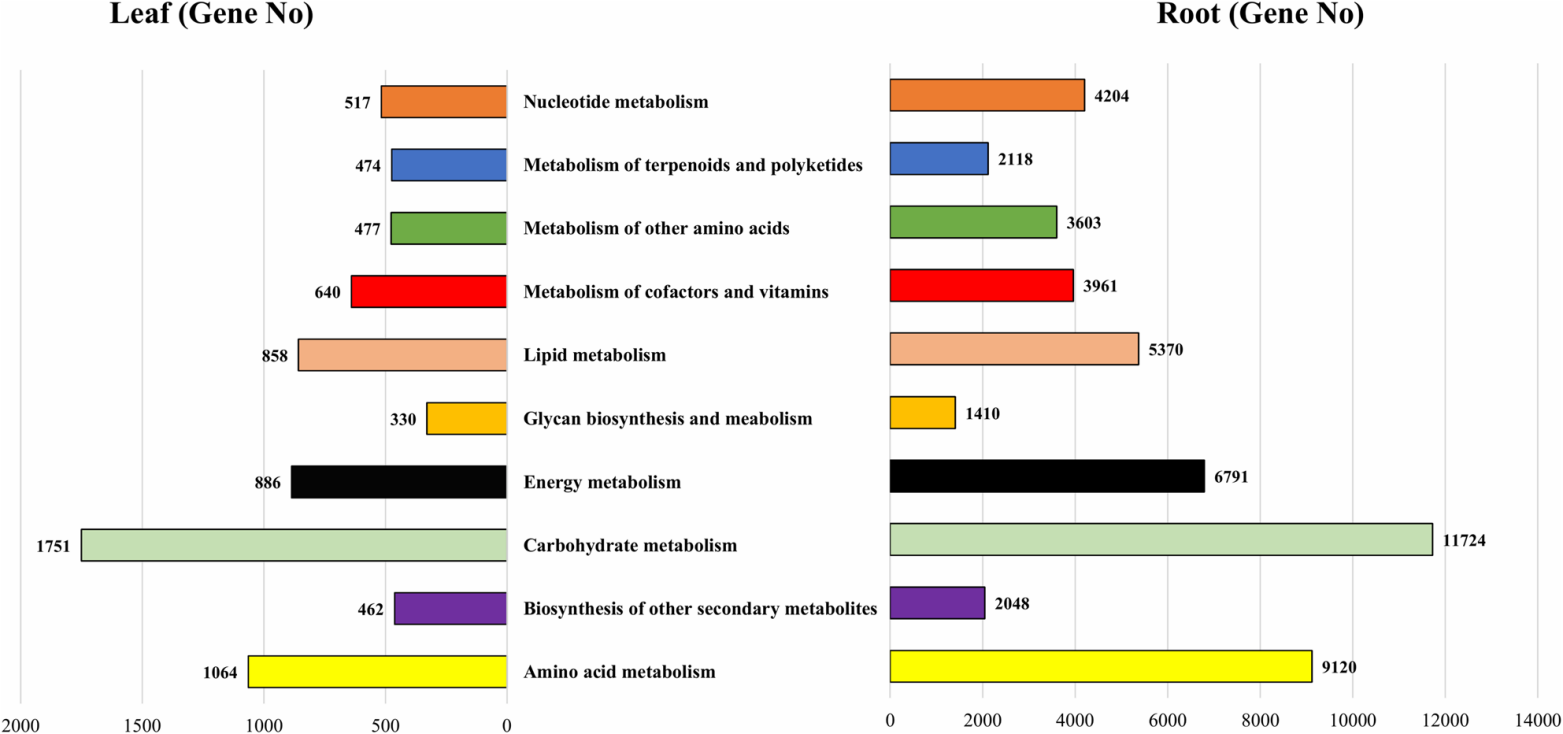
Sub categorization of the metabolism group.

For biological functioning of genes, pathway-based analyses are imperative and this assignment was performed in the KEGG database. In LCL sample, of the 20,506 unigenes, 18,853 were assigned to 5 major groups in KEGG with 26 sub-categories and 131 biochemical pathways (Fig. 5, Supplementary file 4). These major groups were cellular processes (1,004 unigenes), environmental information processing (694 unigenes), genetic information processing (4,285 unigenes), metabolism (8,737 unigenes) and organismal systems (878 unigenes). In these 5 groups, the topmost was the metabolism with 8,737 unigenes, and its further evaluation classified it into 10 subgroups (Fig. 6). Of these, ‘carbohydrate metabolism’ with (1,751) genes involved the highest number of genes, followed by ‘amino acid metabolism’ (1,064) genes and ‘energy metabloism’ with (886) genes (Fig. 6).

**Figure 7:**
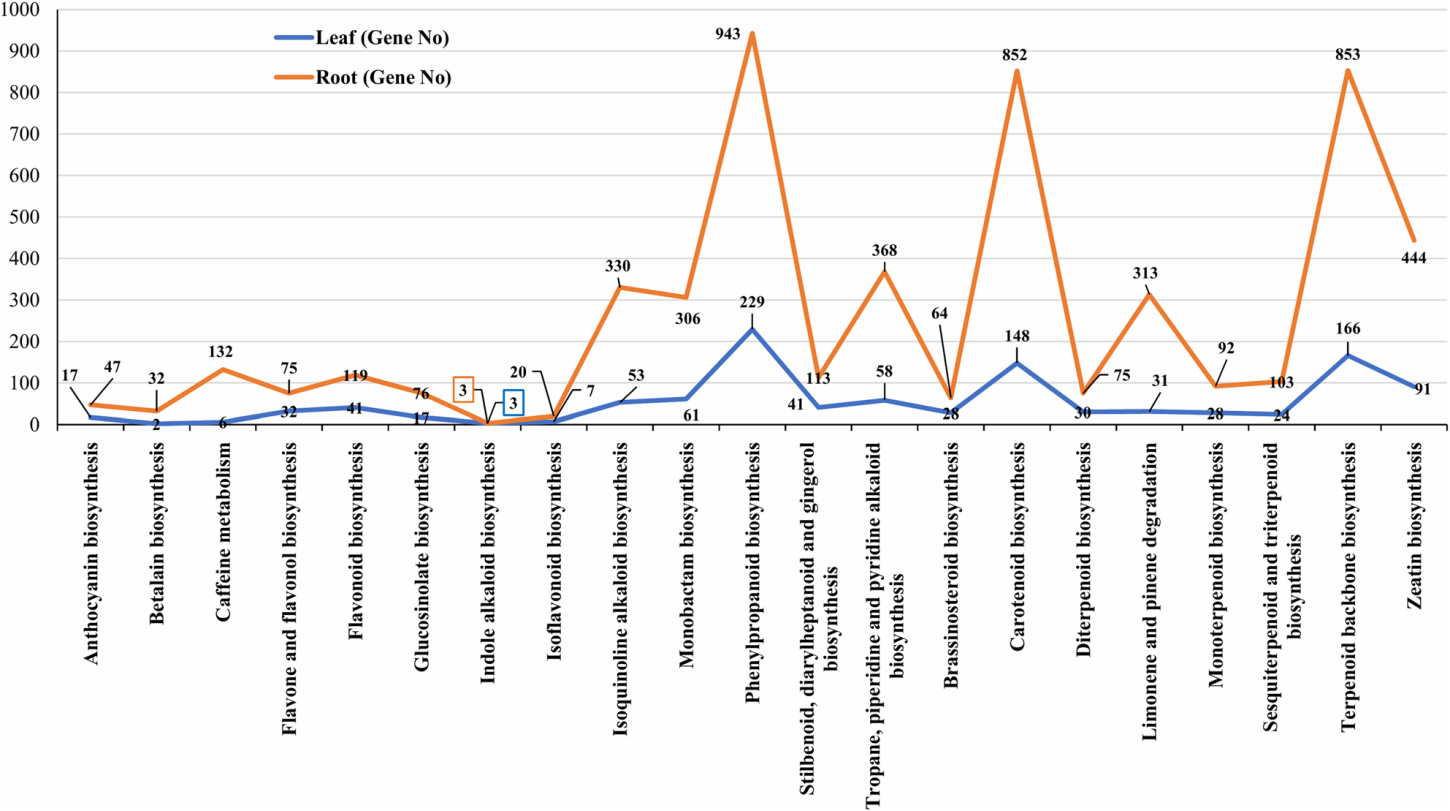
Assigned enzymes function to secondary metabolite pathways with number of genes.

Furthermore, 936 genes were identified that coded for important proteins and have matches with 1,113 enzymes. The function of these enzymes was assigned to 21 secondary metabolite pathways (Fig. 7). Among these pathways 546 genes encoded key enzymes involved in terpeniods biosynthesis including monoterpenoid (28 genes), diterpenoids (30 genes), terpenoid backbone (166 genes), sesquiterpenoid and triterpenoids (24 genes). Similarly, 41 genes were related to flavonoid biosynthesis pathway including flavone and flavonol (32 gene). Among all the secondary metabolite pathways, the phenylpropanoid biosynthesis pathway involved the highest numbers (229) of genes (Fig. 7). These results provide valuable insight into the metabolic pathways in leaves of *L. camara*. These results may provide basis for future studies to identify and characterize genes and transcripts that are involved in important metabolic pathways and may provide insights into the understanding of functions of these genes in the biosynthesis of active compounds in *L. camara*.

Of the 124,076 unigenes in LCR transcriptome, 115,383 were assigned into 5 groups in KEGG with 19 sub-categories and 131 biochemical pathways (Fig. 5, Supplementary file 4). The major groups were cellular processes (7,793 unigenes), environmental information processing (3,384 unigenes), genetic information processing (39,630 unigenes), metabolism (50,349 unigenes) and organismal systems (3,111 unigenes). In these 5 groups, the topmost was the metabolism with 50,349 unigenes was further analysed, which we took for further consideration. The metabolism group was further categorized into 10 subgroups (Fig. 6). In all the 10 sub-groups of metabolism ‘carbohydrate metabolism’ was the highest with (11,724) genes, followed by ‘amino acid metabolism’ with (9,120) genes and ‘energy metabolism’ with (6,791) genes (Fig. 6). Furthermore, 4,166 genes were identified that coded for proteins and have important matches to 5,368 enzymes. The function of these enzymes was assigned to 21 KEGG secondary metabolite pathways (Fig. 7). Among these pathways 2,796 genes encoded key enzymes involved in terpeniods biosynthesis including monoterpenoid (92 genes), diterpenoids (75 genes), terpenoid backbone (853 genes), sesquiterpenoid and triterpenoids (103 genes). The results showed 119 genes were related to flavonoid biosynthesis pathway including flavone and flavonol biosynthesis (75 gene), whereas the highest number of genes (943 genes) among all the secondary metabolite were involved in phenylpropanoid biosynthesis pathway (Fig. 7).

### Phenylpropanoid biosynthesis

**Figure 8:**
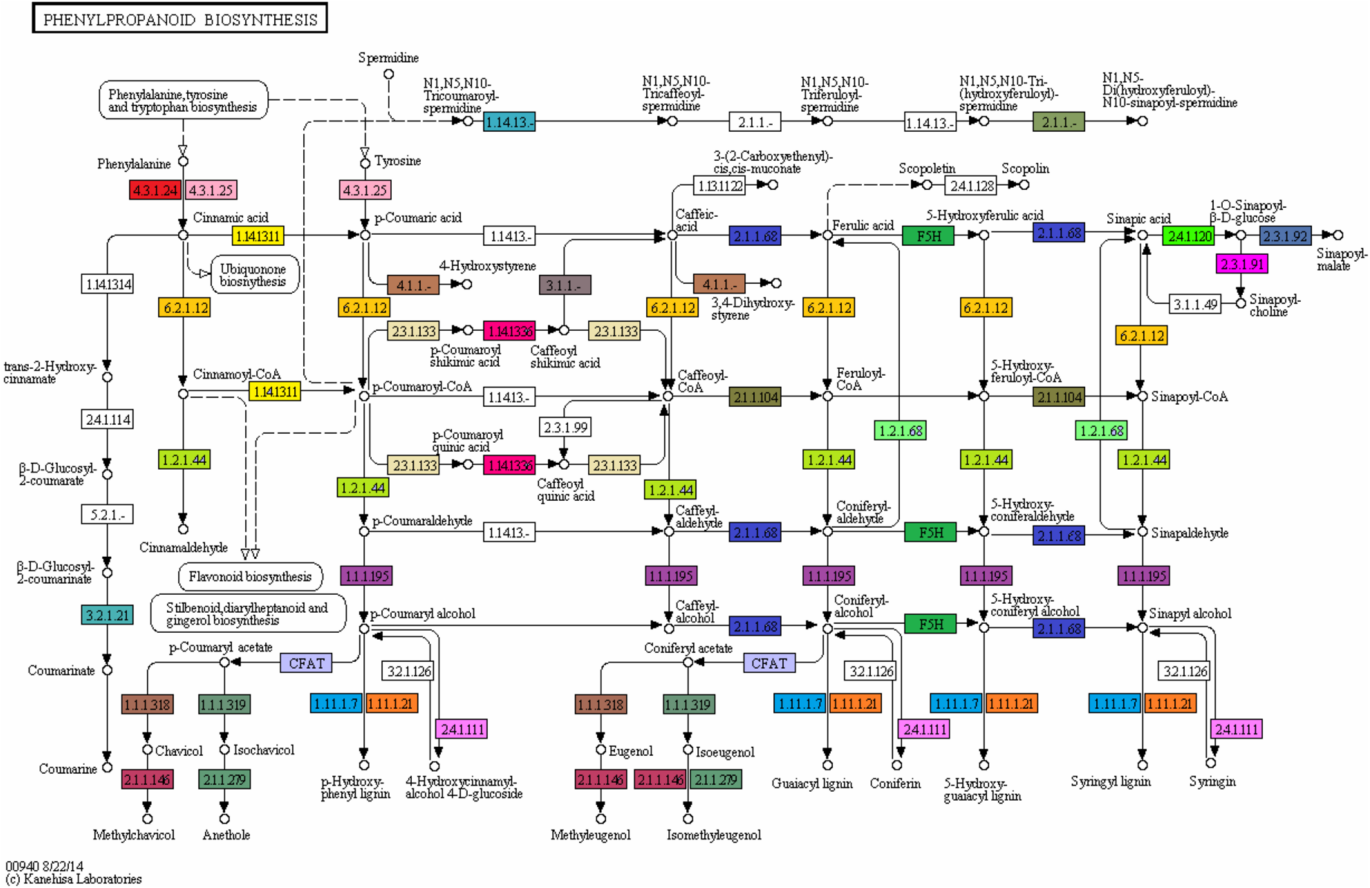
KEGG analysis showing different enzymes identified (one color for each enzyme code or EC) of the Phenylpropanoid biosynthesis pathway. KEGG Pathway ko00940 is adapted from https://www.kegg.jp/kegg/kegg1.html. The KEGG database has been described previously^36,37^.

The current study identified the maximum number of genes for phenylpropanoid biosynthesis pathway and this was taken into further consideration. Phenylpropanoids are phyto-based natural compounds that are usually derived from phenylalanine^48^. Phenylpropanoid plays vital role in plant response to various biotic and abiotic stresses^49^. The process of phenylpropanoid biosynthesis starts with the formation of cinnamic acid from phenylalanine. This cinnamic acid is then converted into cinnamoyl-CoA, p-Coumaryl-CoA, p-coumaryl quinic acid, caffeoyl quinic acid, caffeoyl-CoA, feruloyl-CoA, and sinapoyl-CoA. caffeoyl quinic acid also known as chlorogenic acid, which is a highly soluble phenylpropanoid in Solanaceae and it has been mentioned to play a vital role as an antioxidant and defense molecule^49^. Further, we have identified a total of 229 and 943 genes as well as 17 and 19 enzymes in LCL and LCR transcriptomes respectively and these enzymes are required for phenylpropanoid biosynthesis pathway (Table 4). Our results are different from^50^ where the authors reported only 11 genes from *Solanum trilobatum* that were involved in the biosynthesis of various compounds of this pathway. The important enzymes identified were cinnamyl-alcohol dehydrogenase [EC:1.1.1.195], followed by caffeoyl-CoA O-methyltransferase [EC:2.1.1.104], trans-cinnamate 4-monooxygenase [EC:1.14.13.11], Cinnamoyl-CoA-reductase [EC: 1.2.1.44], phenylalanine ammonia-lyase [EC:4.3.1.24], 4-Coumarate CoA-ligase [EC: 6.2.1.12], and shikimate O-hydroxycinnamoyl-transferase [EC:2.3.1.133]. Interestingly, in root transcriptome two protein coding genes namely cytochrome P450, family 98, subfamily A, polypeptide 8 [EC:1.14.13.-], and Aromatic-L-amino-acid decarboxylase [EC: 4.1.1.28] were identified, that were not detected in leaf transcriptome for phenylpropanoid biosynthesis pathway. The presence of all these enzymes indicates to the phytotherapeutic potential of *L. camara* (Fig. 8, Table 4, Supplementary File 5).

**Table 4:** Important enzymes identified within phenylpropanoid biosynthesis pathway.

Enzyme name	EC number	KO ID	Leaf unigenes no	Root unigenes no
Cinnamyl-alcohol dehydrogenase	1.1.1.195	K00083	17	177
Peroxidase	1.11.1.7	K00430	44	135
Trans-cinnamate 4-monooxygenase	1.14.13.11	K00487	3	12
Caffeoyl-CoA *O*-methyltransferase	2.1.1.104	K00588	9	29
Beta-glucosidase	3.2.1.21	K01188	44	141
4-coumarate-CoA ligase	6.2.1.12	K01904	14	50
Beta-glucosidase	3.2.1.21	K05350	37	99
Cinnamoyl-CoA reductase	1.2.1.44	K09753	5	14
coumaroylquinate(coumaroylshikimate) 3′-monooxygenase	1.14.13.36	K09754	4	16
Ferulate-5-hydroxylase	1.14.-.-	K09755	14	29
Phenylalanine ammonia-lyase	4.3.1.24	K10775	10	35
Peroxiredoxin 6, 1-Cys peroxiredoxin	1.11.1.7	K11188	1	86
Coniferyl-aldehyde dehydrogenase	1.2.1.68	K12355	4	76
Coniferyl-alcohol glucosyltransferase	2.4.1.111	K12356	2	4
Shikimate *O*-hydroxycinnamoyltransferase	2.3.1.133	K13065	12	17
Caffeic acid 3-*O* -methyltransferase	2.1.1.68	K13066	8	16
Caffeoylshikimate esterase	3.1.1.-	K18368	1	5
Cytochrome P450, family 98, subfamily A, polypeptide 8/9	1.14.13.-	K15506	0	1
Aromatic-l-amino-acid decarboxylase	4.1.1.28	K01593	0	1

### Gene expression analysis

**Figure 9:**
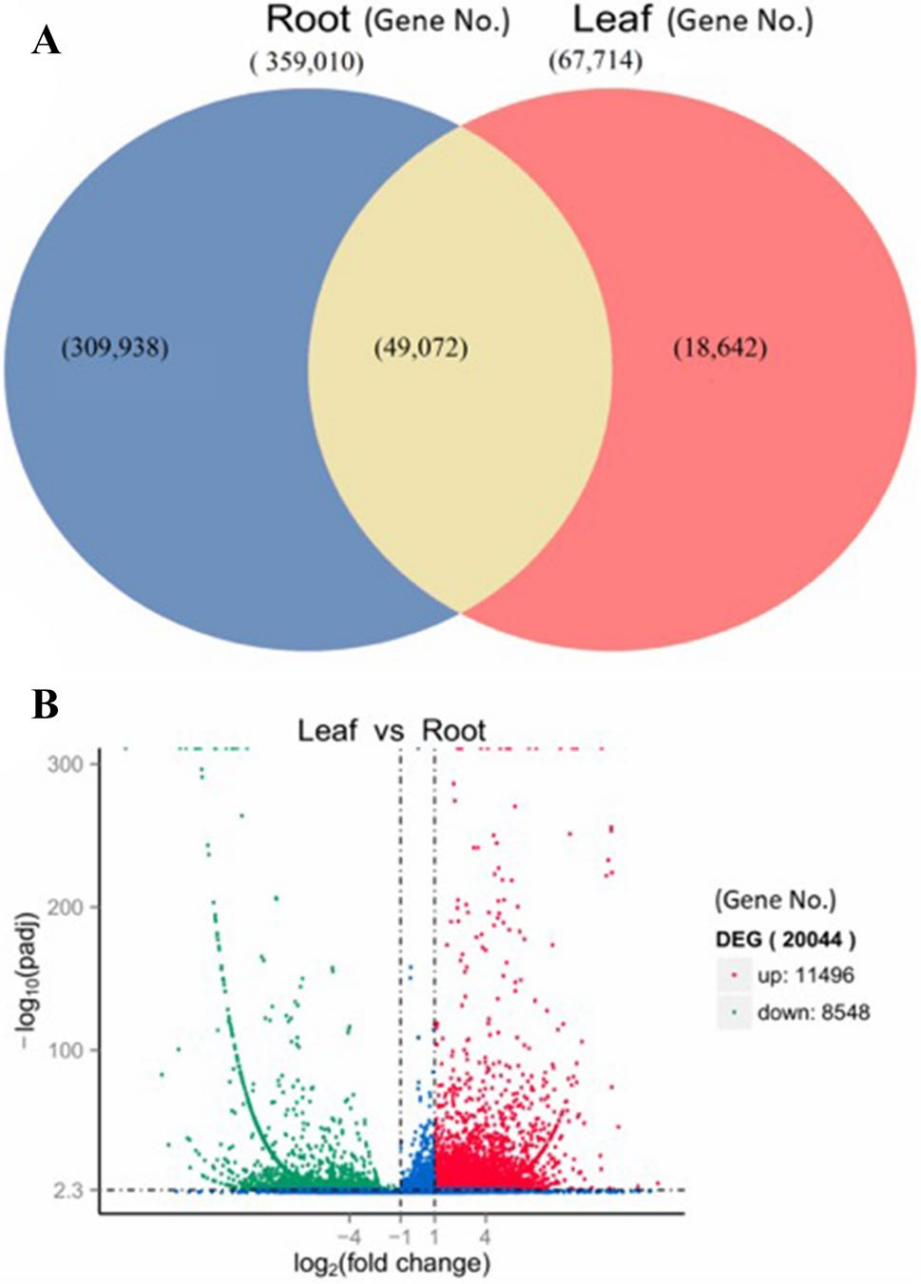
(**A**) Venn diagram showing differentially expressed genes identified in leaf and root of *L.camara*, (**B**) volcano plot showing map of DEGs.

**Figure 10:**
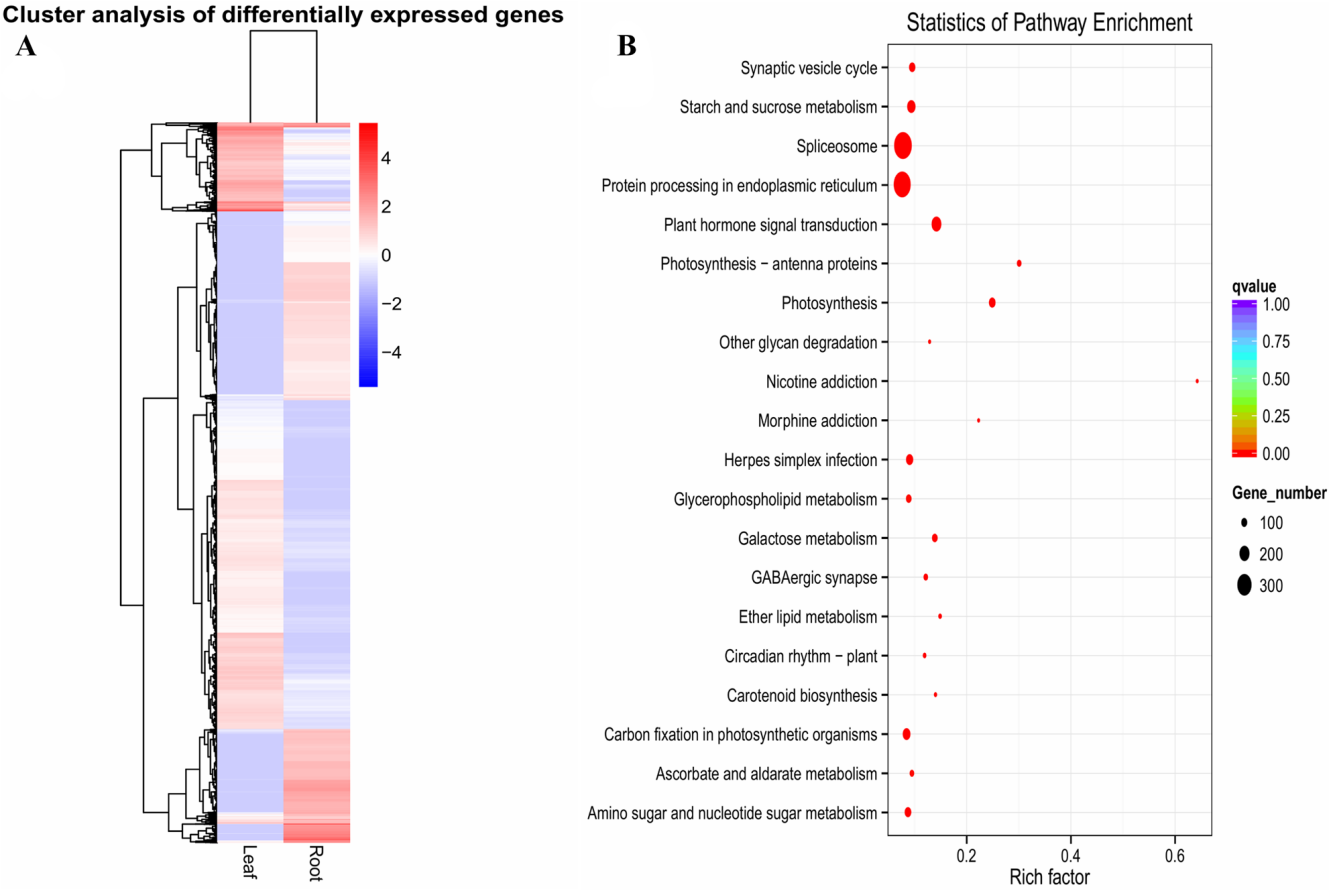
(**A**) Heatmap of upregulated and downregulated genes, (**B**) scatter plot based on number of genes involved in different biological processes.

De novo transcriptome filtered by Corset was used as a reference^51^. RSEM^40^ that map reads back to transcriptome and quantify their expression level was applied. Total reads in gene expression analysis of LCL tissues were 70,155,594, followed by total mapped 54,875,118 (78.22%); whereas total reads in gene expression analysis of LCR tissues were 84,263,224 followed by total mapped 47,187,580 (56.00%). To calculate the gene expression level, RSEM analyzed the mapping results of Bowtie and read count for each gene was converted into FPKM value. In RNA-seq, it is the most common method of estimating gene expression levels, which takes into account the effects of both sequencing depth and gene length on counting of fragments. These results are summarized in Supplementary File 6. Venn diagram, heat map and volcano plot highlights the DEGs and shows genes that are unique and common to leaf and root samples. The total numbers of expressed genes in LCL and LCR tissues were 67,714 and 359,010 respectively, while a total of 49,072 unigenes were found as commonly expressed in both tissues (Fig. 9A). We used DESeq software, to analyze the expression of unigenes in both LCL and LCR transcriptomes by normalizing the values to Fragments Per Kilobase Million (FPKM). The Benjiamini–Hochberg method was used to verify and revise the p-values. Volcano plot demonstrates the fold changes in the expression and statistical comparison (Fig. 9B). A total of 20,044 differentially expressed genes (DEGs) were identified. Within these DEGs 11,496 genes were up regulated and 8,548 genes were down regulated (Fig. 9B). Further, the hierarchical clustering analysis was used to screen the DEGs and cluster them according to their expression in LCL and LCR samples (Fig. 10A). On the basis of pathway enrichment, 20 metabolic and/or biosynthetic processes were predominantly involved. Of these spliceosome and protein processing in the endoplasmic reticulum was comprised by approximately 300 genes (Fig. 10B). Other important processes identified included; synaptic vesicle cycle, starch and sucrose metabolism, plant hormone signal transduction, photosynthesis, carbon fixation in photosynthetic organisms, amino sugar and nucleotide sugar metabolism and galactose metabolism.

## Conclusion

Molecular studies on *L. camara* are rare and high-throughput genotyping efforts are almost non-existent. This study investigated the transcriptomes assembly of *L. camara* leaf and root tissues. A massive data of 70,155,594 and 84,263,224 clean reads were de novo assembled that revealed 72,877 and 513,985 unigenes from leaf and root tissues respectively. Further, the identified unigenes were annotated and functionally characterized in 7 databases. Notably, the numbers of expressed genes in LCL and LCR tissues varied; and 49,072 unigenes were commonly expressed in both tissues. Still, of the 20,044 DEGs 11,496 were up regulated and 8,548 genes were down regulated. Pathway analysis revealed the involvement of 229 and 943 genes (coding for 17 and 19 enzymes) in the biosynthesis of phenylpropanoid pathway in leaf and root tissues respectively. Nonetheless, the genomic resources will not only provide foundation of genomic research in *L. camara* but it will also provide detailed insight into the expression as well as functional analysis, gene cloning and avenues for genomics-assisted breeding in *L. camara*. This study will also serve as baseline to understand the regulation and biosynthesis of crucial bioactive compounds and to select superior alleles/haplotypes of *L. camara* with desired traits in the future.

## Supplementary information

Supplementary Information 1.

Supplementary Information 2.

Supplementary Information 3.

Supplementary Information 4.

Supplementary Information 5.

Supplementary Information 6.
