# Supplementary material for: De novo transcriptome analysis of Lantana camara L. revealed candidate genes involved in phenylpropanoid biosynthesis pathway
Source: Sci Rep. 2020 Aug 13;10:13726. doi: 10.1038/s41598-020-70635-5 (PMC7426850; doi:10.1038/s41598-020-70635-5)

**Supplementary File 1:** RNA leaf and root samples quality report.

***De novo* transcriptome analysis of *Lantana camara* L. revealed candidate genes involved in phenylpropanoid biosynthesis pathway**

Muzammil Shah^1*^, Hesham F. Alharby^1^, Khalid Rehman Hakeem^1^, Niaz Ali^2*^, Inayat Ur Rahman^2,3*^, Mohd Munawar^1^ and Yasir Anwar^1^

*^1^Department of Biological Sciences, Faculty of Science, King Abdulaziz University, Jeddah-21589, Saudi Arabia, ^2^Department of Botany, Hazara University, Mansehra-21300, KP, Pakistan, ^3^William L. Brown Center, Missouri Botanical Garden, P.O. Box 299, St. Louis, MO 63166-0299, USA*

**Corresponding authors e-mail:* [*niazalitk25@gmail.com*](mailto:niazalitk25@gmail.com)*;* [*muzammilshah100@outlook.com*](mailto:muzammilshah100@outlook.com)*; hajibotanist@outlook.com*

1. **QC methodology**

| **Sample Type** | | □ DNA; ^▄^ RNA; □ smRNA; □ Tissue; □ Library ; □ Others |
| --- | --- | --- |
| **Assay Type** | **Preliminary QC** | ^▄^ Agarose Gel Electrophoresis ^▄^ Nanodrop |
|  | **Sample Quantitation** | □ Qubit Fluorometer; □ Agarose Gel Electrophoresis Quantitation; ^▄^Nanodrop |
|  | **Sample Integrity** | ^▄^Agilent 2100; □ Agarose Gel Electrophoresis |
|  | **Sample Purity** | ^▄^ Nanodrop; □ Agilent 2100; □ Agarose Gel Electrophoresis |
|  | **Remarks** | Preliminary QC is compulsory. Only after passing preliminary QC, sample quantitative, sample integrity and purity will be tested. |

**2. QC Results Summary**

| **NO.** | **Sample Name** | **Novogene ID** | **Sample ID** | **Conc (ng/μl)** | **Vol. (μL)** | **Amt.(μg)** | **260/280** | **260/230** | **RIN** | **Conclusion** |
| --- | --- | --- | --- | --- | --- | --- | --- | --- | --- | --- |
| 1 | Leaf | TZTR180906268 | TDO180910725P | 122 | 32 | 3.904 | 2.44 | 0.433 | 7.4 | Pass |

**2.2 Agarose Gel Electrophoresis Results**

**2.2.1 Electrophoresis Condition**

- GelConc.：1% Voltage：180v Run Time：16min

## **2.2.2 Electrophoresis Results Leaf Sample**


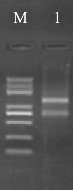


Remark: M:Trans 2K Plus DNA ladder; 1: samples ranged in the order of upper table(1 loaded 1ul).

**2.2.3 The result of Agilent 2100 analysis**


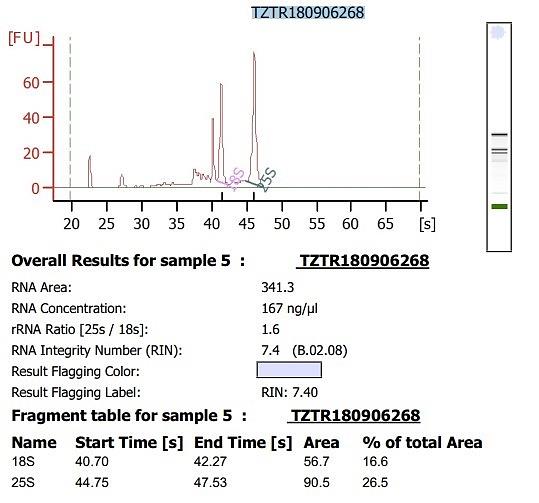


**2.2.4 Electrophoresis result of Root Sample**


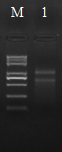


Remark: M:Trans 2K Plus DNA ladder; 1: samples ranged in the order of upper table(1 loaded 1ul).

**2.2.5 The result of Agilent 2100 analysis**


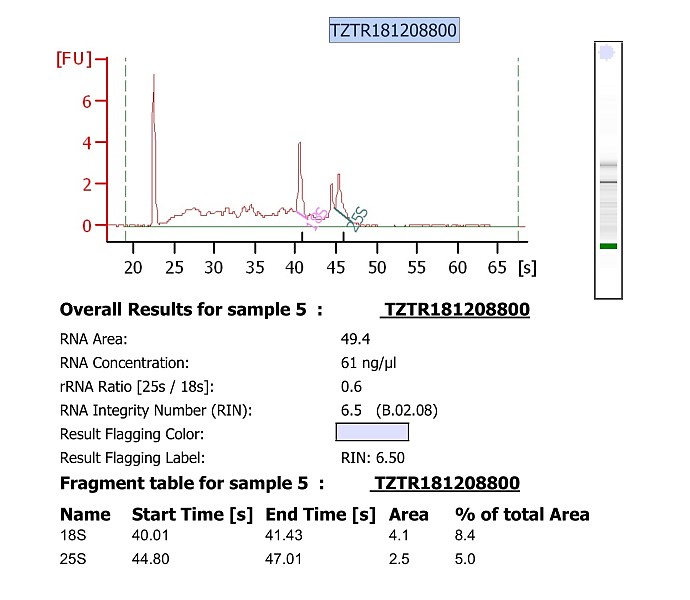

Supplement: Supplementary file 1 — Supplementary Information 1. [file 41598_2020_70635_MOESM1_ESM.docx]
